# The Study of the Frequency Effect of Dynamic Compressive Loading on Primary Articular Chondrocyte Functions Using a Microcell Culture System

**DOI:** 10.1155/2014/762570

**Published:** 2014-04-16

**Authors:** Wan-Ying Lin, Yu-Han Chang, Hsin-Yao Wang, Tzu-Chi Yang, Tzu-Keng Chiu, Song-Bin Huang, Min-Hsien Wu

**Affiliations:** ^1^School of Medicine, Chang Gung University, 259 Wen-Hwa 1st Road, Gueishan Township, Taoyuan County 333, Taiwan; ^2^Department of Orthopaedic Surgery, Chang Gung Memorial Hospital, Linko, 5 Fusing St., Gueishan Township, Taoyuan County 333, Taiwan; ^3^Graduate Institute of Biochemical and Biomedical Engineering, Chang Gung University, 259 Wen-Hwa 1st Road, Gueishan Township, Taoyuan County 333, Taiwan; ^4^Graduate Institute of Chemical and Materials Engineering, Chang Gung University, 259 Wen-Hwa 1st Road, Gueishan Township, Taoyuan County 333, Taiwan

## Abstract

Compressive stimulation can modulate articular chondrocyte functions. Nevertheless, the relevant studies are not comprehensive. This is primarily due to the lack of cell culture apparatuses capable of conducting the experiments in a high throughput, precise, and cost-effective manner. To address the issue, we demonstrated the use of a perfusion microcell culture system to investigate the stimulating frequency (0.5, 1.0, and 2.0 Hz) effect of compressive loading (20% and 40% strain) on the functions of articular chondrocytes. The system mainly integrates the functions of continuous culture medium perfusion and the generation of pneumatically-driven compressive stimulation in a high-throughput micro cell culture system. Results showed that the compressive stimulations explored did not have a significant impact on chondrocyte viability and proliferation. However, the metabolic activity of chondrocytes was significantly affected by the stimulating frequency at the higher compressive strain of 40% (2 Hz, 40% strain). Under the two compressive strains studied, the glycosaminoglycans (GAGs) synthesis was upregulated when the stimulating frequency was set at 1 Hz and 2 Hz. However, the stimulating frequencies explored had no influence on the collagen production. The results of this study provide useful fundamental insights that will be helpful for cartilage tissue engineering and cartilage rehabilitation.

## 1. Introduction

Articular cartilage is a tough and resilient connective tissue lying at the end surface of long bones near joints. The biological function of cartilage to provide resistance to mechanical loading is primarily attributed to its abundant extracellular matrix (ECM), mainly consisting of glycosaminoglycans (GAGs) and collagen [1]. The homeostasis of the ECM is maintained by the articular chondrocytes, the only cell species existing in articular cartilage. Upon damage, adult cartilage has a limited ability of self-repairing. To treat cartilage defects, cartilage tissue engineering is generally regarded as a promising method. The common approach to cartilage tissue engineering involves the* in vitro* cultivation of tissue constructs by using (1) chondrogenic cells, (2) scaffolds capable of providing a three-dimensional (3D) structure for tissue development, and (3) bioreactors capable of providing the suitable extracellular conditions necessary for the cells to regenerate functional cartilaginous tissues [2]. Although proof of principle has been demonstrated [1], few* in vitro* cartilage tissue engineering investigations have generated an appropriate tissue that meets the functional demands placed upon this tissue* in vivo*.

One of the key technical hurdles to functional cartilage tissue engineering is the lack of fundamental understanding of the link between extracellular conditions and chondrocyte functions. Though several studies have been carried out to find out the relationship between articular chondrocyte and environment, the majority of them have focused on exploring the effect of biochemical factors on cellular functions [3]. Physical factors like mechanical stimulation have been reported to play an important role in modulating chondrocyte physiology and, in turn, cartilage homeostasis [4]. Although some fundamental investigations have been carried out to probe the effect of mechanical stimulation on articular chondrocyte physiology [1, 5–11], the relevant studies are, to some extent, not comprehensive. The possible cause behind this is a lack of appropriate experimental tools, by which the effect of mechanical stimulation on chondrocyte function can be quantitatively determined in an efficient, precise, physiologically meaningful, and cost-effective manner.

The effects of mechanical loading on articular chondrocytes are complex. Under physiological mechanical stimulation, the articular chondrocytes in cartilage are subjected to complicated physical events including hydrostatic pressure, tensile stress, shear stress, and compressive stress [11]. Among these events, compressive stimulation plays an important role in modulating the articular chondrocyte functions [1]. Several devices or bioreactors have been developed for investigating the effect of compressive stimulation on articular chondrocyte physiology. These cell or tissue culture apparatuses commonly make use of the direct compression on the chondrocytes-embedded constructs or on cartilage explants due to its similarity to* in vivo* loading by intra-articular contact [1]. According to the working regimen, the cell or tissue culture devices capable of providing compressive loading to cells can be divided into two categories, namely, static [5] and dynamic systems [1, 6–11]. Dynamic compressive stimulation is predominantly adopted because dynamic loading is physiologically meaningful [1, 12]. Numerous cell or tissue culture devices, capable of generating dynamic compressive stimulation, have been demonstrated to be feasible for providing compressive loading to the cartilage explants, or chondrocytes-embedded scaffold [1, 5–10]. Nevertheless, these devices are normally technically demanding, complicated, large, and costly, which could limit the experimental throughput. Moreover, most of these devices adopt static cell culture format [1, 5–7, 9, 10, 13], where the culture medium is literally supplied in a manual and batch-wise manner. This could lead to high contamination risk and fluctuating culture condition [14]. The latter could hamper the precise quantification of the link between the mechanical stimulation and articular chondrocyte functions since reports in literature have demonstrated that the articular chondrocytes are fairly sensitive to extracellular environments [15].

**Figure 1:**
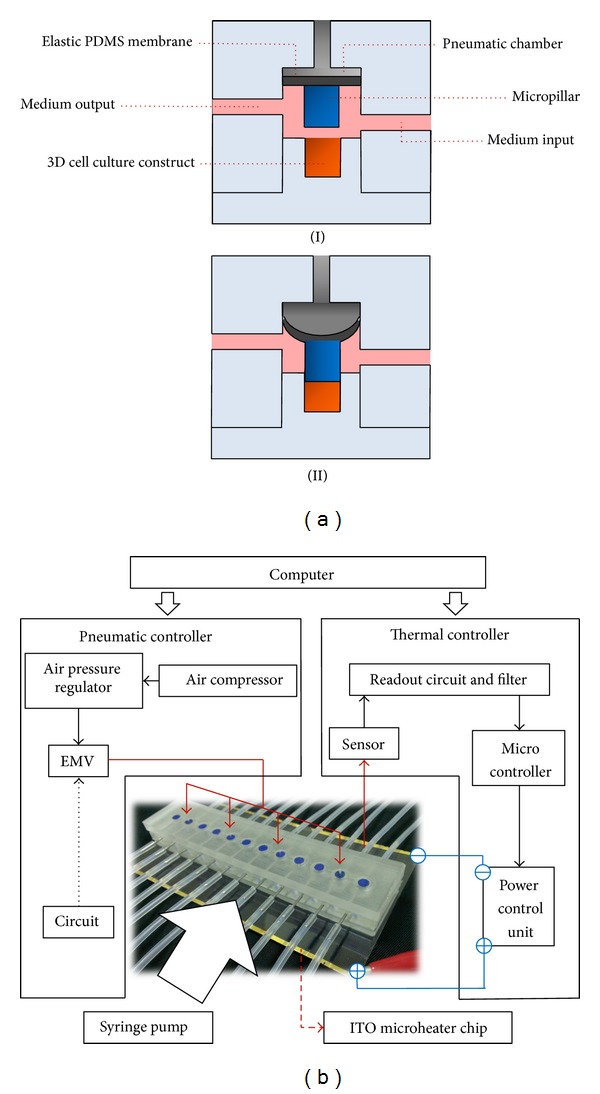
(a) Schematic illustration of the pneumatically-driven compressive loading mechanism: (I) the rest state and (II) the pneumatically-driven deformation of an elastic PDMS membrane leading to the vertical movement of the connected micropillar that in turn generates compressive loading onto the 3D cell culture construct below (cross-sectional view) (a video clip is provided as a Supplementary Material available online at http://dx.doi.org/10.1155/2014/762570), and (b) the photograph of the microcell culture system, and the schematic illustration of overall experimental setup (EMV: electromagnetic valves; ITO: indium tin oxide).

While tissue engineering scientists are making progress [16], they might need an appropriate device to aid their research. To address the issue, a microcell culture system capable of providing dynamic compressive loading to 3D cell culture constructs was proposed in our previous study (Figure 1(a)) [11]. One of its distinctive features is the function of tunable compressive loading generation. The mechanism is based on the pneumatically driven deformation of an elastic polydimethylsiloxane (PDMS) membrane, which in turn exerts compressive loading onto a 3D cell culture construct through a micropillar (Figure 1(a)) [11]. By modulating the frequency and pressure of the input pneumatic conditions, the compressive loading can be generated in a controllable manner. Other technical features of the microcell culture system are (1) low cost, (2) high throughput, (3) low consumption of research resources due to its small scale, (4) maintaining a stable and well-defined culture condition due to the perfusion culture format and miniaturized culture scale [14, 17], (5) efficient and precise sample (e.g., cells) loading, and (6) easy operation. Borrowing from our previous experience on the micro cellculture system, the frequency (0.5, 1.0, and 2.0 Hz) effect of dynamic compressive loading (strain: 20 and 40%) on the cell viability and proliferation as well as the metabolic and biosynthetic activities of primary articular chondrocytes were investigated.

## 2. Materials and Methods

### 2.1. The Microcell Culture System and Experimental Setup

The microcell culture system is composed of 12 individual microbioreactors (D: 7.5 mm; H: 6.5 mm). At the bottom of each microbioreactor, there is a cylindrical cavity (D: 1 mm; H: 2.3 mm; volume: 1.8 *μ*L), which is not only used to accommodate a cell/biomaterial scaffold for 3D cell culture but also to quantitatively define the volume of such sample loading. The overall sample-loading process was described previously [11].

For high throughput perfusion cell culture purpose, each microbioreactor was designed to perfuse with its own separate medium supply through silicon tubing driven by a multichannel syringe pump (KDS 220, KD Scientific Ltd., USA). In addition, the microcell culture platform was placed on the surface of a transparent indium-tin-oxide- (ITO-) based microheater chip to provide a stable thermal condition of 37 ± 0.2°C for cell culture [18]. To pneumatically drive and control the compressive loading mechanism, 4 via holes were connected with 4 air tubes from a custom-made pneumatic controller. Within the hand-held controller, an air compressor (MDR2-1A/11, Jun-Air Inc., Japan), 4 electromagnetic valves (EMV) (S070M-5BG-32, SMC Inc., Taiwan), and a programmable control circuit were integrated to activate and to control the generation of compressive loading. The overall experimental setup is illustrated in Figure 1(b).

### 2.2. The Exertions of Dynamic Compressive Loading: Operating Conditions and Long-Term Stability

**Figure 2:**
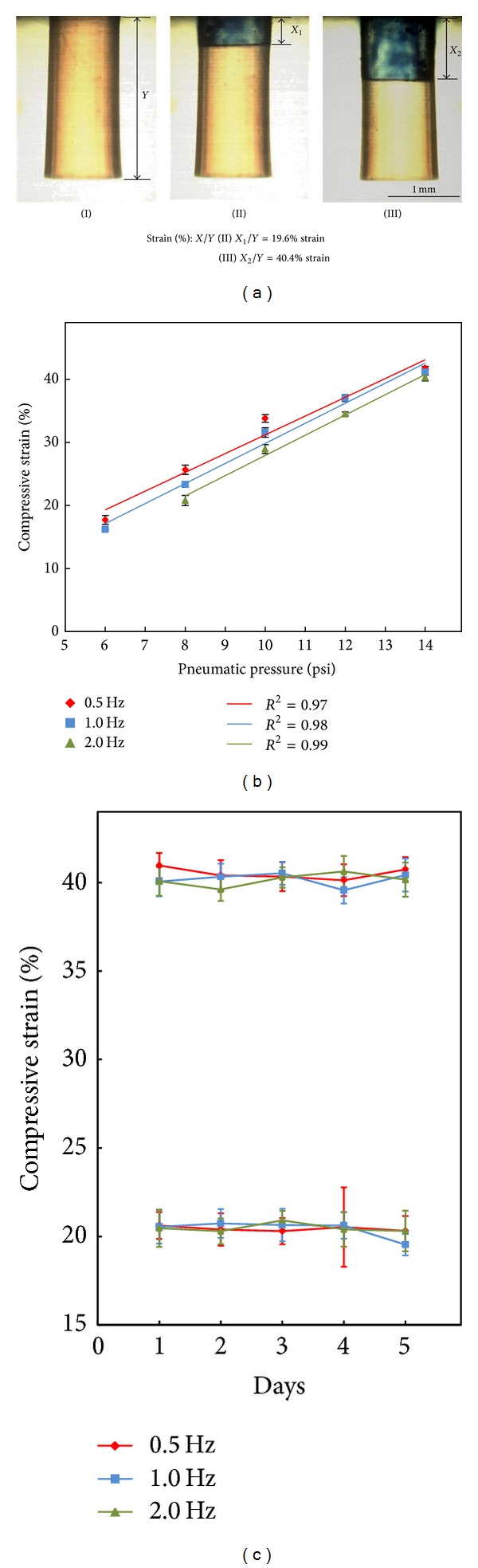
(a) Experimental quantification of the generated compressive strain by capturing the action images of micropillar using a high-speed CCD. The compressive strain (%) is measured by the equation: *x* (deformation length)/*y* (height of 3D cell culture construct; the photograph (I))∗100%. Photographs (II)-(III) showed the observed deformation lengths of 0.45 mm (*X*_1_) and 0.93 mm (*X*_2_) with the corresponding compressive strain of 19.6% and 40.4%, respectively. (b) The quantitative relationship between the compressive strain and the pneumatic pressure applied under different operating frequency conditions (0.5, 1.0, and 2.0 Hz). (c) Evaluation of long-term stability of the generated compressive strain (lower and higher level: 20% and 40%, resp.) under the operating frequencies of 0.5, 1.0, and 2.0 Hz.

In this study, the operating stimulating frequencies (0.5, 1.0, and 2.0 Hz) were manipulated through the programmable control circuit and EMVs. For the magnitude of compressive loading, it was modulated by tuning the magnitude of the pneumatic pressure exerted in the pneumatic chamber (Figure 1(a)) through an air pressure regulator (Figure 1(b)). It was expressed as compressive strain (the ratio of compressive deformation per unit length along the compressive axis) and quantified by the method reported previously [11]. Briefly, the dynamic deformation of a 3D cell culture construct was experimentally observed with the aid of a high-speed charge-coupled device (CCD) camera (MC1311, Mikrotron, Germany). In the measurements, the sequential action images of the dynamic deformation of a 3D cell culture construct were captured at the resolution speed of 100 frames sec^−1^. The compressive strain (%) was then measured by the equation: *x* (the deformation length)/*y* (the height of 3D cell culture construct)∗100%.  Figure 2(a) showed the microscopic images of the 3D cell culture constructs subjected to the dynamic compressive loading with magnitudes of 20% and 40% strain. Based on above approaches, the quantitative relationships between the magnitude (6–14 psi) and the frequency (0.5, 1.0, and 2.0 Hz) of the pneumatic pressures exerted and the resulting compressive strain (%) were experimentally determined. In this work, furthermore, the dynamic compressive stimulation was subjected to chondrocytes-encapsulating 3D culture constructs for up to 5 days. In order to examine the long-term stability of dynamic compressive loading, the daily measurements of the compressive strain (20% and 40%) generated under different operating frequencies (0.5, 1.0, and 2.0 Hz) were carried out, based on the approach aforementioned.

### 2.3. Perfusion 3D Articular Chondrocyte Culture under Different Dynamic Compressive Loading

Primary articular chondrocytes were isolated from the femorotibial joint as previously described [3]. The cell suspension thus obtained was assessed microscopically for cell number and viability using 0.4% (w/v) trypan blue in phosphate buffered saline (PBS; Invitrogen, Taiwan). Only cell preparations with cell viability greater than 95% were then used. In this study, the primary articular chondrocytes were encapsulated in alginate hydrogel to form a 3D cell culture construct. Briefly, the isolated cells were then immobilized in 6% (w/v) alginate hydrogel at a cell density of 2.4 × 10^7^ cells mL^−1^. The mixture was subsequently loaded into the cylindrical cavity in the microbioreactors (Figure 1(a)). After sample loading and device assembling, each microbioreactor was perfused with 0.5 M calcium chloride solution at a flow rate of 1 *μ*L min^−1^ for 25 min for the solidification of the alginate hydrogel. This was followed by perfusion articular chondrocyte culture under a serial compressive loading settings (strain: 20% and 40%; operating frequency: 0.5, 1.0, and 2.0 Hz; daily regimen: three consecutive cycles of 1 hour loading and 1 hour relaxation) for up to 5 days using the established experimental setup (Figure 1(b)). In this research, Dulbecco's Modified Eagle's Medium (DMEM) (with 1000 mg L^−1^ glucose, 25 mM HEPES, without sodium bicarbonate; unless stated otherwise all chemicals were purchased from Sigma, Taiwan) supplemented with 10% foetal bovine serum (Invitrogen, Taiwan), 2% antibiotic/antimycotic solution, and 50 *μ*g mL^−1^ ascorbic acid was continuously perfused to the individual microbioreactors at a flow rate of 60 *μ*L hr^−1^. After a 5-day perfusion cell culture under compressive stimulation, the cell viability was observed microscopically through fluorescent staining. In addition, both culture medium and 3D cell culture constructs were collected to evaluate the proliferative, metabolic, and biosynthetic activities of articular chondrocytes by measuring the cellular DNA, lactic acid, GAGs, and hydroxyproline levels, respectively.

### 2.4. Bioassays

#### 2.4.1. Evaluation of Cell Viability and Proliferation

The viability of the articular chondrocytes in alginate hydrogel under different compressive stimulation was evaluated using a fluorescent dye kit (LIVE/DEAD Viability/Cytotoxicity Kit L-3224, Molecular Probes). After cell culture, briefly, the remaining culture medium inside the microbioreactor chambers was removed. 10 *μ*L of the dye reagent containing 1 *μ*M calcein and 2 *μ*M ethidium homodimer-1, prepared according to the manufacturer's instruction, was loaded into each microbioreactor chamber. After 20 min incubation, the images of live (green) and dead (red) cells were captured using a confocal microscope (LSM 510 META, Zeiss, Germany). Cell viability was then quantified by counting the live (green) and dead (red) cells using a software program (SimplePCI version 5.2.1, Compix Inc., PA, USA) [19]. In addition, the cell proliferation of articular chondrocytes was evaluated by quantifying the DNA contents of cells. The DNA content of cells was detected according to Hoemann et al. [20]. Calf thymus DNA (5 to 125 ng) was used as standard.

#### 2.4.2. Lactic Acid

The lactic acid produced and released into culture medium was measured using a Lactate Reagent Kit (Trinity Biotech Plc., Ireland) [21]. The assay was carried out as directed by manufacturer's instructions. A lactate solution at a concentration of 50~500 mg L^−1^ made from dissolving lactate sodium salt in deionised water (DI) water was used as standard.

#### 2.4.3. Glycosaminoglycans (GAGs) and Hydroxyproline

A colorimetric reaction based on Farndale et al. [22] was used to detect GAGs. The method used in this experiment was modified according to Hoemann et al. [20]. Absorbance at 540 nm was read using a microplate reader (Sunrise, Tecan Ltd, Taiwan) and chondroitin sulphate. A sodium salt in the range between 0.01 *μ*g and 1.25 *μ*g was used as standard. Moreover, total collagen production was assayed as hydroxyproline content after hydrolysis. The hydroxyproline content of insoluble collagen was determined according to Stegemann and Stalder [23]. In this evaluation, the hydroxyproline was assayed by a modified method by Urban and McMullin [24].

### 2.5. Statistical Analysis

Data from at least three separate experiments were analyzed and presented as mean ± standard deviation. For a given experiment, each condition was tested in triplicate (*n* = 9). One-way ANOVA analysis with a statistical significance level of 0.05 was used to examine the effects of different compressive stimulation on the proliferative, metabolic, and biosynthetic activities of chondrocytes after 5 days of culture. The Tukey Honestly Significant Difference (HSD) post hoc test was used to compare the differences between two compressive loading conditions when the null hypothesis of ANOVA analysis was rejected.

## 3. Results and Discussions

### 3.1. Effect of Operating Conditions on the Generation of Dynamic Compressive Loading

In this study, a pneumatically-driven membrane-based actuation scheme was adopted to create compressive loading on 3D cell culture constructs (Figure 1(b)). This working mechanism has also been utilized in a wide variety of micropumps in various microfluidic systems [25, 26]. Different from the other approaches to generate such mechanical movements, pneumatically-driven mechanism is generally regarded to have lower fabrication cost and simpler fabrication and operation process [25, 26]. In the microcell culture system, the magnitude of the pneumatic pressure exerted in the pneumatic chamber (Figure 1(a)) and its operating frequency are two key parameters that can manipulate the dynamic compressive loading on a 3D cell culture construct.  Figure 2(b) shows the quantitative relationships between the operating conditions and the resulting compressive loadings. For a given operating frequency, the generated compressive strain increased proportionally (*R* ^2^: 0.97, 0.98, and 0.99 for the 0.5, 1.0, and 2.0 Hz, resp.) with the increase of the applied pneumatic pressure within the experimental conditions investigated (Figure 2(b)). Neglecting the mechanical resistance of 3D cell culture construct, the above finding is due to the fact that the resultant compressive strain is directly proportional to the deformation magnitude of PDMS membrane. The key moving component in the mechanism of compressive loading generation is the elastic PDMS membrane and its connected micropillar (Figure 1(a)). The pneumatically-driven deformation of a PDMS membrane is well described as follows [27]:

(1) $$\begin{array}{c} w_{o}=\frac{pa^{2}}{4\sigma_{o}h}, \end{array}$$

where *w*_*o*_, *p*, *a*, *σ*_*o*_, and *h* represent the maximum magnitude of membrane deformation, applied pneumatic pressure, radius of PDMS membrane, intrinsic tensile stress, and thickness of membrane, respectively.

From above equation, it can be observed that the deformation magnitude of a PDMS membrane is proportional to the magnitude of pneumatic pressure applied. These could reasonably explain the proportional relationship between the applied pneumatic pressure and the resultant compressive strain as observed in Figure 2(b). Moreover, it was also found that the generated compressive strain decreased with the increase of operating frequency (Figure 2(b)). At a given pneumatic pressure, the dynamic process to load a pneumatic chamber with an air pressure is time-dependent [28, 29]. Higher operating frequency implies shorter imposition time (e.g., 2.0, 1.0, and 0.5 sec round^−1^ for the 0.5, 1.0, and 2.0 Hz, resp.) of pneumatic pressure input. This could accordingly lead to reduced PDMS membrane deformation and thus the lower level of compressive strain is generated. The experiments also found that the maximum frequency and strain that can be obtained in the system are 4 Hz and 50% stain, respectively. In the study, furthermore, a dynamic compressive stimulation was applied onto an articular chondrocytes-encapsulating 3D culture construct for 5 days. For a more accurate investigation, the long-term stability of the compressive loading was experimentally examined. This issue has been generally ignored in several studies based on the same pneumatically-driven membrane-based actuations [25, 26, 28].  Figure 2(c) exhibited the long-term variations of the compressive strain generated under different operating conditions. For the lower compressive strain range of around 20%, the measured compressive strains were found to have no significant difference (*P* > 0.05, ANOVA) during the 5 days operation under a given operating frequency tested. Furthermore, when the compressive strain generated under different operating frequencies were compared, they were measured to have no statistical difference (*P* > 0.05, ANOVA) at each time point investigated during the 5-day operation. Similar results were also found at the higher compressive strain range of about 40%. Taken together, the above findings might indicate that the proposed mechanism for dynamic compressive loading generation was capable of providing a stable compressive loading during the cell culture period.

### 3.2. Effect of Dynamic Compressive Loading on the Cell Viability, Proliferation, and Metabolic Activities of Articular Chondrocytes

**Figure 3:**
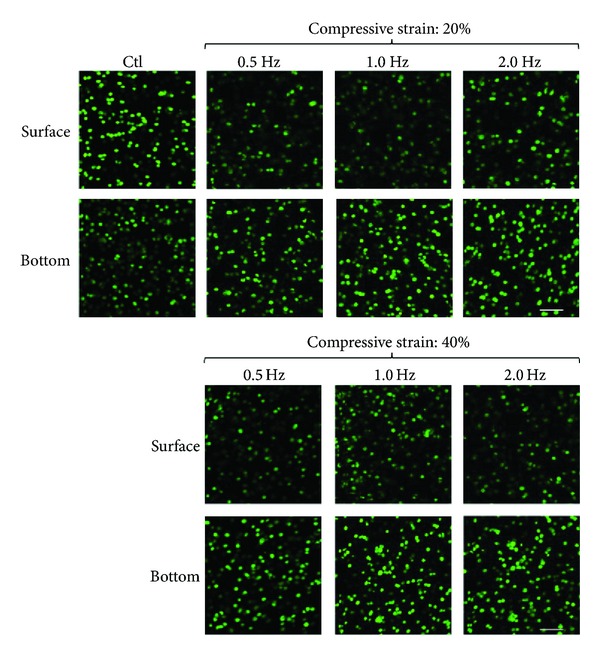
Microscopic observation of cell viability after 5 days of cell culture under different compressive stimulation conditions (strain magnitudes: 20% and 40%; stimulating frequencies: 0.5, 1.0, and 2.0 Hz) using the live/dead fluorescent dye. Green and red dots represent live and dead cells, respectively. The upper and lower images show the cells at the surface and the bottom of the 3D cell culture constructs, respectively.

In this study, the primary articular chondrocytes cultured in 3D alginate hydrogel constructs were subjected to various compressive stimulation conditions (strain: 20%, and 40%; frequencies: 0.5, 1.0, and 2.0 Hz) for up to 5 days. In addition, the dynamic compressive stimulation regimen was based on 3 consecutive cycles per day, (one cycle is defined as 1 hour of loading followed by 1 hour of relaxation), which was reported to be more physiologically relevant [1, 30, 31]. In this study, the impact of the compressive stimulation on the articular chondrocyte viability was observed using a fluorescent dye staining and quantified using an image analysis.  Figure 3 showed the microscopic images of the articular chondrocytes treated with different compressive stimulation conditions, in which the green and red dots represent the live and dead cells, respectively. Overall, the articular chondrocytes cultured under dynamic compressive loading kept the average cell viability as high as 95%, without statistical difference (*P* > 0.05, ANOVA) among the experimental conditions tested. These outcomes were consistent with the previous findings showing that the cell viability of articular chondrocytes is not mechanically affected by the compressive strain being lower than 51.5% [11]. Articular cartilage is a tissue designed to withstand compression during joint movement, and thus the articular chondrocytes are normally subjected to a wide range of mechanical loadings* in vivo*. In normal physiological conditions, a human articular cartilage is subjected to the compressive strain ranging from 20% to 30%* in vivo* [1, 32, 33]. In this study, the higher compressive strain around 40% is regarded as the excessive loading of articular cartilage during vigorous exercise [33]. Taken together, the above findings could indicate that the articular chondrocytes might keep high cell viability under normal physiological condition or even under the excessive compressive strain as high as 40%–50%.

**Figure 4:**
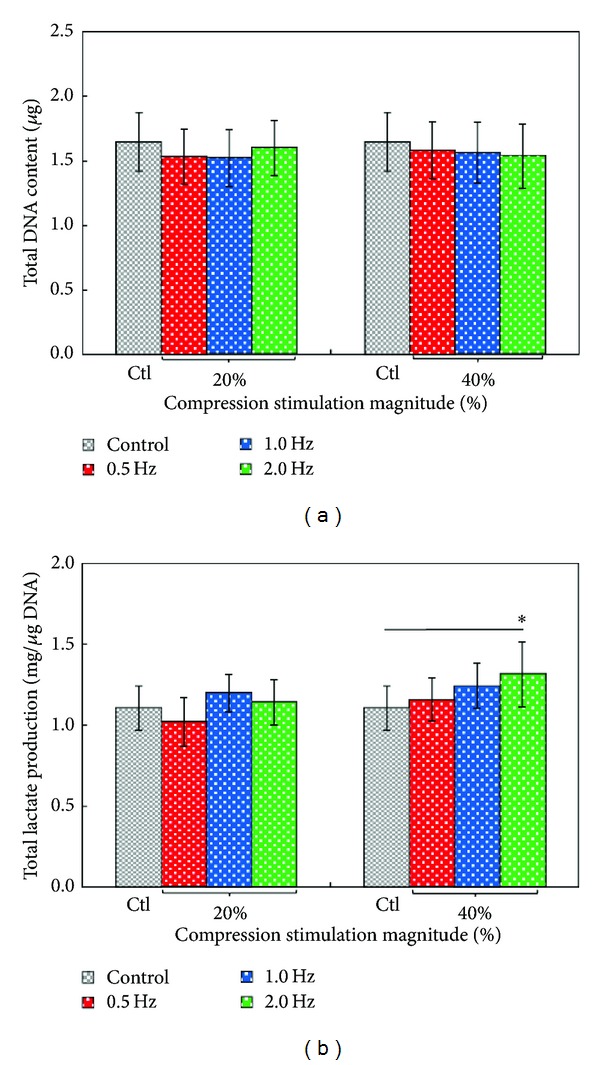
The evaluation of the (a) proliferative (DNA content measurement) and (b) metabolic (lactate production measurement) activities of the primary articular chondrocytes under different dynamic compressive loading conditions (as indicated) after 5 days of culture. The results are displayed as mean ± standard deviation of 3 separate experiments (*n* = 9). Significant differences are expressed as *(*P* < 0.05).

Furthermore, the DNA content of cells was measured as an indicator of articular chondrocytes proliferation after 5 days of cell culture in this study. As shown in Figure 4(a), there was no statistical difference (*P* > 0.05, ANOVA) between the control (no compressive stimulation) and the mechanical compression-treated cases, meaning that the stimulating frequency (0.5, 1.0, and 2.0 Hz) and the magnitude (20%, and 40% strain) of compressive stimulation play no role in the proliferation of articular chondrocytes within the experimental conditions investigated. The articular chondrocytes, accounting for less than 5% of tissue volume, are the only cell species existing in articular cartilage. It has been reported that articular chondrocytes could not significantly proliferate when they are cultured in 3D culture environment, whereas their proliferative activity might increase while cultured in 2D monolayer format [34]. The phenomenon of no positive effect on articular chondrocyte proliferation found in this study is also discovered in several primary articular chondrocyte 3D cultures no matter if they are treated with mechanical stimulation [8, 9] or not [16].

In addition, the metabolic activities of articular chondrocytes treated with various ranges of compressive loading were evaluated by measuring the lactic acid production. Regarding articular chondrocytes metabolism, anaerobic glycolysis is the major metabolic pathway [35], from which the lactic acid is the main metabolic product. Therefore, the production of lactic acid was utilized to indicate the chondrocytes metabolic activity in this study. Within the experimental conditions explored, results (Figure 4(b)) revealed that the stimulating frequency of compressive loading did not significantly affect the metabolic activities of chondrocytes under the strain magnitude of 20%. At the higher strain magnitude of 40%, however, the frequency of compressive stimulation did have significant impact on the metabolic activities of articular chondrocytes (*P* < 0.05, ANOVA). The total lactic acid production of chondrocytes cultured under the stimulating frequency of 2 Hz (40% strain) was 18.77% statistically higher than that of the control case (*P* < 0.05). Nevertheless, the lactic acid production was not statistically different among the cells experiencing stimulating frequency lower than 2 Hz. The dynamic compression at the 40% strain and 2 Hz is close to the compressive condition that human articular cartilage experiences during high intense exercise [33, 36]. The finding disclosed in this study could indicate that the high frequency vigorous exercise might upregulate the metabolism of articular chondrocytes in comparison with the case without compressive stimulation.

### 3.3. Effect of Dynamic Compressive Loading on the Biosynthetic Activities of Articular Chondrocytes

**Figure 5:**
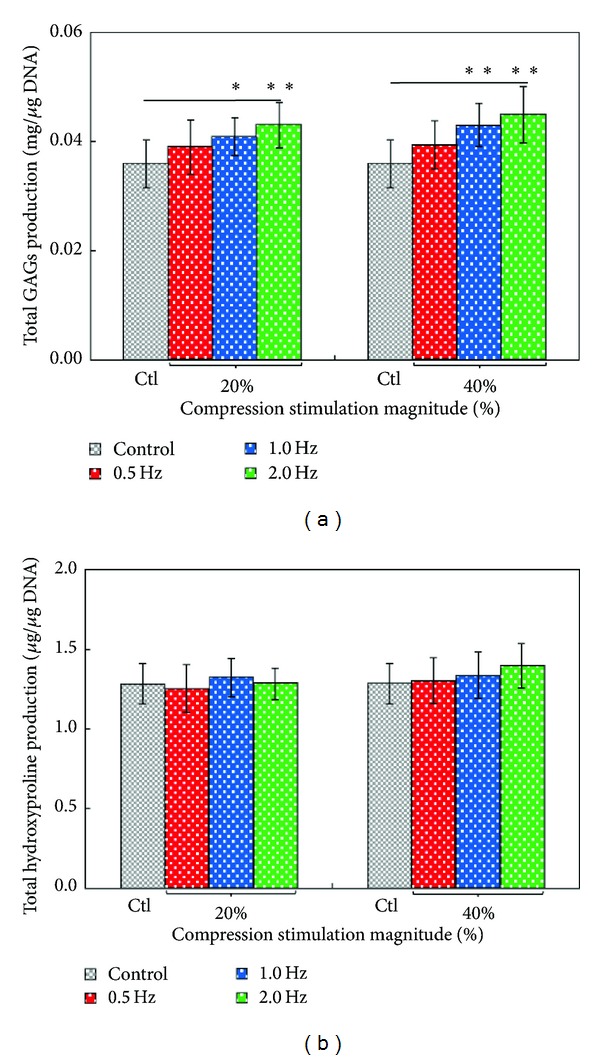
The evaluation of the biosynthetic activities ((a) GAGs and (b) hydroxyproline production) of the primary articular chondrocytes under different compressive loading conditions (as indicated) after 5 days of culture. The results are displayed as mean ± standard deviation of 3 separate experiments (*n* = 9). Significant differences are expressed as *(*P* < 0.05) and **(*P* < 0.01).

Under compressive stimulation, several* in vitro* investigations revealed that the articular chondrocytes could rearrange their synthesis of ECM components [1, 5–11]. However, few of them have reported the role of stimulating frequency on the biosynthetic activities of articular chondrocytes [5, 6, 9]. In this study, results (Figure 5(a)) exhibited that the stimulating frequency could significantly influence the GAGs synthesis of articular chondrocytes (*P* < 0.05, ANOVA) both at the two strain magnitudes (20% and 40%) explored. In the ECM of articular cartilage, the GAGs are mainly responsible for the compressive stiffness of such tissue [10, 37]. It is reported that articular chondrocytes may synthesize more GAGs in responding to dynamic compressive loading [38]. For the two compressive strain groups tested, the total GAGs synthesis of articular chondrocytes cultured under the stimulating frequencies of 1 Hz, and 2 Hz were statistically (*P* < 0.05 for 1 Hz (20% strain) and *P* < 0.01 for 2 Hz (20% strain) and 1 and 2 Hz (40% strain)) higher than that of the control case. Nevertheless, such phenomenon was not found among the cells experiencing the stimulating frequency lower than 0.5 Hz. The stimulating frequencies of 1 Hz and 2 Hz are about the frequency range of compressive loading that the human articular cartilages are subjected to during walking [39] and running [36] conditions, respectively. Therefore, the findings revealed in Figure 5(a) might imply that the GAGs biosynthesis of articular chondrocytes might be upregulated under these physiological conditions. In terms of collagen biosynthesis of chondrocytes, results (Figure 5(b)) demonstrated that the formats of compressive loading including stimulating magnitude and frequency did not significantly (*P* > 0.05, ANOVA) influence the total hydroxyproline production within the experimental conditions investigated.

As a whole, the above findings demonstrated that the GAGs synthesis was mechanically upregulated while the collagen production was not affected when 3D cultured chondrocytes were subjected to dynamic compressive stimulation. Reports in literature have also revealed similar research outcomes. For example, some studies have disclosed that dynamic compressive or tensile loading might increase the GAGs biosynthesis of articular chondrocytes but these mechanical stimulations were found to have no positive effect on the synthesis of collagen [5, 8, 11, 21, 38]. Nevertheless, other studies have exhibited different results [1, 6, 31, 37]. For example, some studies have reported that dynamic compression has advantageous effect both on the production of GAGs and collagen [1, 6, 31]. Moreover, Hunter et al. [37] disclosed the adverse effect of dynamic compressive stimulation on GAGs and collagen production of articular chondrocytes. The reasons behind the above discrepancies are complicated. It is well accepted that the regimen of compressive stimulation [31], as well as the scaffolding material [9, 37], can influence the ECM synthesis of articular chondrocytes. In this study, the daily regimen of dynamic compressive stimulation was based on 3 consecutive cycles of 1 hour loading and 1 hour relaxation, which was reported to be more physiologically relevant [1, 30, 31]. In the investigations, moreover, the primary articular chondrocytes were microencapsulated in an alginate construct. In addition to its excellent biocompatibility [40–42], alginate hydrogel is a commonly used scaffolding material for cartilage tissue engineering because the phenotypic or functional stability of articular chondrocytes in this material can be maintained [43–45]. Furthermore, mammalian cells are sensitive to extracellular microenvironments [16]. To precisely study the cellular responses to extracellular conditions, a stable and homogenous culture environment is crucial because it can provide a well-defined and quantifiable culture condition. Unlike the most of cell culture models (e.g., the static or larger scale cell culture devices [1, 5–7, 9, 10, 13]) adopted for the similar investigations, one of the technical highlights in this study is the use of a perfusion-based microscale cell culture platform capable of providing dynamic compressive stimulation to cells. It enables scientists to create more stable and well-controlled culture environments due to the continuous nutrient supply and waste removal [14, 17] and the phenomenon of low chemical gradients existing in 3D cell culture construct [14], respectively. As a whole, this study has utilized the microcell culture system to mainly explore the stimulating frequency effect of compressive loading on the articular chondrocytes functions. The use of the microcell culture system for more systematic researches will be required to reconcile the differences with data acquired through conventional methods.

## 4. Conclusions

In this study, the stimulating frequency (0.5, 1.0, and 2.0 Hz) effect of compressive loading (strain: 20 and 40%) on the cell viability and proliferation as well as the metabolic and biosynthetic activities of articular chondrocytes were investigated. It was found that the dynamic compressive loadings explored in this study did not have significant impact on the articular chondrocyte viability and proliferation, in which the cells kept the cell viability as high as 95%. As to the metabolic activity of articular chondrocytes, the frequency of compressive stimulation did have significant influence when the compressive strain was increased to 40%, in which the lactic acid production of cells treated with the stimulating frequency of 2 Hz (40% strain) was 18.77% statistically higher than that of the case without mechanical stimulation. This compressive loading is close to the condition that human articular cartilage experiences during high intense exercise. For the biosynthetic activities of articular chondrocytes, results showed that the GAGs synthesis was mechanically upregulated when the stimulating frequency of compressive loading was set at 1 Hz and 2 Hz. These are about the frequency range of compressive loading that the human articular cartilages subjected to during walking and running conditions, respectively. Nevertheless, the formats of compressive loading including stimulating magnitude and frequency did not have significant impact on the collagen production of articular chondrocytes within the experimental conditions explored. As a whole, the research findings above are found fundamentally important both for articular cartilage tissue engineering and articular cartilage rehabilitation.

## Supplementary Material

The video clip demonstrated the dynamic process of compressive loading (1 Hz, 30% strain) working onto the 3D cell culture constructs (light-yellow colored) within the microcell culture system (composed by 3 individual microbioreactors in this video clip).
